# Supplementary material for: Are carbon emissions trading and green financial instruments synergistic? -Comprehensive quantitative research based on content analysis
Source: PLoS One. 2024 Mar 7;19(3):e0298601. doi: 10.1371/journal.pone.0298601 (PMC10919601; doi:10.1371/journal.pone.0298601)
Supplement: S1 Data — (ZIP) [file pone.0298601.s001.zip › Data/地级市Z_F工作报告.zip!/║╙▒▒/▓╫╓▌╩╨/└┤╫╘╣1⁄2╓┌║┼í╛┬φ┐╦╩2╛▌═°í┐_2004.docx]

各位代表：

现在，我代表市人民政府，向大会作工作报告，请予审议，并请各位政协委员和其他列席会议的人员提出意见。

2003年政府工作回顾

2003年，是全面贯彻落实党的十六大精神的第一年，也是新一届政府的起始年。我们在省委、省政府和市委的正确领导下，坚持以邓小平理论和“三个代表”重要思想为指导，全面贯彻党的十六大精神，团结和带领全市人民解放思想，开拓创新，团结拼搏，奋发进取，克服了“非典”带来的严重不利影响，圆满完成了市十一届人大一次会议确定的各项工作任务，全市国民经济和社会发展实现了新的突破，为进一步加快发展奠定了坚实的基础。

一、国民经济稳定健康快速发展，势头良好

（一）各项经济指标快速增长。2003年，全市国内生产总值预计完成621.8亿元，增长12.1%，高出计划2.1个百分点。其中，第一产业增加值95.9亿元，第二产业增加值320.1亿元，第三产业增加值205.8亿元，分别增长9.6%、13.2%和11.3%。全市入统工业增加值完成97.8亿元，利税完成30.5亿元， 分别增长21.4%和34%，分别高出计划11.4和24个百分点。全社会固定资产投资完成178.5亿元，增长23.6%，其中技改投资完成16.6亿元，增长23.4%。全部财政收入完成56.4亿元，增长6.4%，其中一般预算收入完成23.3亿元，增长10.6%。

（二）对外开放取得新进展。全市新批“三资”项目合同54个，增长50%，投资总金额2.8亿美元，增长3.8倍，合同外资额9950万美元，增长2.7倍。三资企业注册资本1.4亿美元，增长3.1倍，实际完成外资8404万美元，其中，直接利用外资3027万美元，完成省下达的招商引资任务。同时加强与京津及周边地区的经济技术合作，在劳务输出、农副产品和建筑建材产品销售等方面取得新成效。坚持外资、外贸、外经一起抓，全市完成出口总值2.98亿美元，增长34.7%，“从大运河走向渤海湾”的开放观念深入人心，对外开放的紧迫感进一步增强。

（三）民营经济势头强劲。坚持舆论上引导，政策上支持，环境上保护，努力为民营经济提供更加适宜的生存环境，创造更加广阔的发展空间，使全市民营经济总量和实力进一步增强。到2003年底，民营经济完成增加值365亿元，营业收入1270亿元，实现利润102亿元，上缴税金18亿元，分别增长16.4%、16.8%、14%和13.6%。

（四）项目建设全面突破。全市投资和项目建设，呈现出前所未有的良好局面。项目个数明显增加 。投资1000万元以上的重点项目946个，总投资1255亿元，分别比上年增加487个和781亿元。亿元以上大项目达到198个，比上年增加115个，是近年来最多的一年。 新开工项目、竣工项目个数比上年增加一倍还多，其中，投资1000万元以上的项目完成投资87.3亿元，同比增加36.1亿元。黄骅港二期工程、黄骅港杂货码头、液体化学品码头、40万吨PVC等一大批有影响的重点项目在全市人民的期待中开工建设，进展顺利。

（五）企业改革稳步推进。以产权制度改革为核心，进一步加大了国有企业改革力度，全市674家市、县属国有企业中，已有649家完成改制，改制面为96.3%。继续实施“帮危解困”工程，全市100家危困企业中，已有69家实现脱困，其中，市直42家已有29家进入实质性运作阶段，核销呆坏帐5.4亿元，盘活闲置资产3.5亿元，解除担保3.9亿元。商贸流通企业改革同时展开。中捷、南大港农场行政管理体制改革已经到位，为沧州经济快速发展注入了新的活力。

（六）发展环境不断优化。坚持把优化发展环境作为第一竞争点，实行了环境建设“一票否决”制度，在政府部门全面实施承诺践诺、首办负责、投诉受理、责任追究等制度；深入开展民主评议行风活动，召开了优化环境万人广播电视大会，对多起破坏经济环境的典型案件做了公开处理；行政审批事项在两次削减的基础上，再次削减了578项。硬环境建设，完成了沧州市域城镇体系规划和沧州市区总体规划修编；投资13.8亿元，实施了一批交通、排水、污水处理等基础设施项目，其中完成交通建设工程244项，总投资9.7亿元，增长43.7%。大浪淀水库实现了封闭管理，确保了城市用水安全。全市上下，环境意识渐入人心，环境建设渐入佳境。

（七）“三农”工作成效显著。围绕增加农民收入这一历史性课题，在确保粮食生产稳定增长的前提下，以“四大产业、一大工程”为重点，强龙头、扩基地，大力发展优势农产品生产，农业投入产出效益稳步提高。全市粮食总产260.8万吨，棉花总产10.5万吨，分别比去年增加1.7万吨和3万吨；植树造林面积71.8万亩，创历史最高；新增牧草面积43万亩，牧草加工能力由14.5万吨提高到58万吨；无公害蔬菜发展到80万亩，总产309万吨；草业、枣业协会正式组建，50家产业化龙头企业不断壮大，4家畜牧类龙头企业通过了ISO9000体系认证，8个产品申报了省级以上名牌。海兴县与香港南华集团合作的“农业示范园区”开始建设，沧县巨龙枣业枣制品、泊头鸭梨等产品打入了位居世界500强榜首的连锁配送企业——沃尔玛。

二、社会事业全面发展，民主法制和精神文明建设进一步加强

（一）科教文体等社会事业不断发展。举办了“沧州市首届科技成果暨技术交易会”，与大专院校、科研单位签订合作协议17项。实施高新技术产业化项目23项，其中，宝丰线缆“一步法硅烷交联电缆”项目达到国际先进水平。新建学校112所，另有68所已完成主体工程，危险校舍改造和教育布局调整居全省前列。高教、职教、成教均取得新进展，卫校升专新校区建设已经开工。群众文化活动红红火火，歌词《晒盐的汉子》荣获全国“五个一”工程奖。竞技体育成绩有了新的提高，群众性健身活动空前活跃。统计、审计、档案、物价、残联、气象、地震、人防、广播电视、民族宗教等各项工作都取得了新的成绩。

（二）可持续发展能力不断增强。从严控制人口数量，全市人口自然增长率控制在5.65‰以内。加强国土资源、海洋资源保护，基本农田保护区面积71.9万公顷，保护率85.7%。认真实施《海河流域污染防治“十五”规划》，加大对重点污染区域的治理力度。市污水处理厂、垃圾处理厂等工程开工建设，取缔“十五小”重污染企业101家。

（三）民主法制和精神文明建设进一步加强。各级政府自觉接受同级人大及其常委会的监督，充分听取政协和各民主党派、工商联、无党派人士及专家学者、人民群众的意见和建议，认真办理人大代表建议和政协提案，积极支持工会、共青团、妇联、科协等人民团体开展工作。坚持科学民主决策，邀请专家、学者对我市海洋经济发展战略进行了深入研讨。全面推进依法行政。加强社会治安综合治理，社会秩序进一步好转。扎实推进精神文明建设，广泛开展以社会公德、职业道德、家庭美德为主要内容的公民道德建设活动，全社会思想道德素质不断提高。民兵预备役、国防教育等活动进一步开展，并荣获“全国双拥模范城”称号。

（四）人民生活水平进一步提高。新增就业岗位4.2万个，新增就业4.93万人，其中下岗失业人员再就业2.1万人。全市城镇登记失业率为3%，低于省4.5%的指标。实现了“两个确保”，基本生活保障率、养老金按时足额发放率和社会化发放率均达到100%。以养老、失业和基本医疗三大保障为主的社会保障体系逐步健全和完善。加大扶贫攻坚力度，全面落实各项救灾措施。全年发放救灾款1507万元，灾区群众和弱势群体的基本生活得到有效改善。新修乡村公路451.3公里，为236个村解决了饮水困难。城市居民人均可支配收入6728元，农民人均纯收入2691元，分别增长9.2%和5.6%。

三、夺取了抗击“非典”斗争的阶段性胜利

去年4月，面对突然遭遇“非典”疫情的严竣形势，我们在中共沧州市委的领导下审时度势，沉着应对，一切从确保人民群众的生命安全和身体健康出发，一切从沧州的实际出发，一切从抗击“非典”的需要出发，超前预见，超前谋划，科学决策，果断指挥，及时实行群防群控、联防联控工作机制，建立了市、县、乡、村四级防控网络，加大封堵、排查、隔离、诊疗等关键环节工作力度，有效控制了非典疫情的输入和传播。在同“非典”抗争的日日夜夜，广大干部将个人的生死荣辱置之度外，心中只有人民群众。不止一个人像曹连才那样，不惜倒在抗击“非典”的阵地上，用自己的血肉之躯铸成保护人民群众生命安全的盾牌。而广大群众则充分理解政府的所作所为，哪怕暂时伤害了自己的利益，也坚定地团结在政府周围。白衣战士，可歌可泣，干群同心，共赴危难。经过70多天惊心动魄的较量，我们胜利地保卫了沧州、保护了沧州人民，创造了治愈率百分之百、零感染、零死亡、持续无疫情天数最长等4项指标的全省第一，得到省委、省政府的充分肯定和全市人民的高度赞扬。然而，在抗击“非典”斗争中我们收获的绝不仅仅是这“四个第一”，而是难以估量的精神财富，它使我们的人民群众认识到：在事关生死存亡的关键时刻，我们的政府是信得过的、是靠得住的、是真心实意为人民的政府。它同时也使我们的各级政府认识到：只要我们真心实意为人民服务，只要我们真正代表人民群众的利益，人民群众就会坚定不移地跟着我们走。

在夺取抗击“非典”的胜利之后，我们又遭遇了罕见的风暴潮灾害。沧海横流，方显英雄本色。在翻江倒海一般的袭击面前，全市人民再一次紧密团结在中共沧州市委的旗帜下，万众一心，战胜灾害，不仅把损失降到最低限度，而且没死一个人。在不断战胜灾害和困难的过程中，不仅锻炼了干部、凝聚了人心、密切了干群关系，我们的各项事业也在灾害和困难的洗礼中不断发展、不断前进！

2003年的成绩，来之不易。认真总结回顾，有四点深刻的体会：一是必须时刻不忘人民群众的利益。只有忠实地践行执政为民宗旨，把提高人民生活水平作为执政之本、为政之要，时刻注意维护人民群众的利益，真正做到情为民系、权为民用、利为民谋，才能取信于民，增强向心力和战斗力，只有当人民群众需要我们的时候，切实感受到我们的存在，当我们需要人民群众的时候，人民群众才会坚定地同我们站在一起。二是必须将政府工作置于党的领导之下。去年的工作给了我们许多启示，而最重要的启示之一，就是党的领导是我们战胜困难、夺取胜利的根本保证。面对复杂多变的情况，中共沧州市委作为一个坚强的领导集体，处变不惊，沉着应对，对政府工作给予了及时、正确的指导，坚定、周到的支持，使得全市各项工作即使在最紧张的情况下，始终有条不紊。实践再次让我们深刻体会到，政府工作必须始终置于党委的领导之下。重要工作，要及时向党委请示汇报，对于党委的决策和意图，则要自觉地在政府工作的实践中体现出来，只要党的领导得以顺利实施，党政军民步调一致，建设新沧州的进程中尽管充满困难，但没有什么困难可以阻挡住我们前进的步伐！三是必须树立正确的政绩观。我们反对“形象工程”和所谓的“政绩工程”，并不是不要形象，更不是不要政绩，而是不要不顾实际情况，不惜劳民伤财的政绩，不要经不起实践检验的政绩，不要给后任留下后遗症的政绩。我们提倡脚踏实地，力戒空谈，想群众之所虑、急群众之所难、谋群众之所求，在发展沧州和不断提高人民群众生活水平上有作为，出政绩，真正推动一方发展、惠及一方百姓，所作所为经得起时间的冲刷和历史的检验。四是必须牢记执政兴市的第一要务。不管遇到什么困难和挫折，不管面对什么气候和变数，只有牢牢把握发展这个第一要务，才能增强我们的实力，缩小与发达地区的差距，才能解决诸多历史遗留问题，不断提高和改善人民群众的生活，才能使我们在任何情况下都保持清醒的头脑，不随风转向，不人云亦云，不贻误事业，不坐失良机。我们坚信，只要我们毫不动摇地坚持把发展作为第一要务，就一定能够充分调动一切积极因素，凝聚合力，克服前进道路上的一切艰难险阻，实现振兴沧州、发展沧州、繁荣沧州的宏图大业！

各位代表，过去的一年，的确是不平凡的一年，虽然困难比预想的大，但我们取得的成绩更比预想的好。这些成绩的取得，首先是全市各级领导机关、领导干部认真学习并践行“三个代表”重要思想，高扬“树正气、讲团结、求发展”的主旋律，带领广大群众抢抓机遇，苦干实干的结果；其次是在中共沧州市委的领导下，沧州风清气顺、政通人和，人大依法监督、政协参政议政，干部队伍精神振奋、人民群众意气风发，为经济和社会发展提供了最基本也是最根本的保证；三是历届政府勤政为民、谋求发展，为沧州奠定了坚实的基础，一旦时机到来，蓄积的能量便喷涌而出。面对这春潮涌动、万马奔腾的大好局面，我们一定要发扬成绩，戒骄戒躁，百尺竿头，更进一步，把我市经济和社会发展推向一个更高的层次。在此，我代表市人民政府，向辛勤工作在各条战线上的全市各族人民，向给予我们支持与监督的人大代表、政协委员，向大力支持我们工作的驻沧单位、人民解放军和武警部队，以及所有关心、支持沧州发展的各界人士，表示崇高的敬意和衷心的感谢！

在认真总结工作、肯定成绩的同时，我们也清醒地看到前进中存在的困难和问题，主要是：经济总量仍然偏小，特别是工业规模小，结构性矛盾依然突出，对全局起支撑和拉动作用的产业不多，新能源、新材料、信息技术和生物工程方面的企业更少；农业基础薄弱，产业化水平不高，农民增收缓慢；财政紧张状况得不到根本性缓解，地方可用财力有限，致使许多该办的事情暂时还无能为力；城镇化水平低，劳动力供需矛盾突出，城乡就业压力增大，部分群众生活困难；经济发展环境还不尽如人意，个别地方社会治安形势不好，安全生产的基础不牢固，政府工作人员官僚主义、形式主义和行政效率不高的问题仍然存在，等等。对此，我们将采取切实有效的措施，认真加以解决。

2004年主要工作和措施

2004年是完成“十五”计划的关键一年，是共和国建国55周年，也是实现沧州大发展、快发展的希望之年。扬长避短，趋利避害，开创性地做好今年的工作，是时代赋予我们的使命，也是全市680万人民的热切期盼。

2004年政府工作的指导思想是：以党的十六大精神和“三个代表”重要思想为指导，认真贯彻落实十六届三中全会和省委六届四次全会精神，高扬“树正气、讲团结、求发展”的主旋律，坚持以人为本，树立全面、协调、可持续的发展观，突出大发展、快发展这一主题，正确处理改革、发展、稳定的关系，以全方位加快对外开放统揽经济工作全局，紧紧围绕结构调整，大力度加快项目建设，放手壮大民营经济，深化企业改革，优化经济发展环境，加强民主法制建设、政治文明建设和精神文明建设，促进全市经济和社会的协调发展，推动全市经济和社会事业跨上一个新台阶。

2004年主要任务目标：国内生产总值增长11.5%；全社会固定资产投资增长16.5%；全部财政收入增长10%，其中一般预算收入增长9%；直接利用外资增长65.2%，外贸出口增长12.5%；城镇居民人均可支配收入增长7%，农民人均纯收入增长4%；人口自然增长率控制在6.54‰以内。同时，完成十二件大事、实事和十二项民心项目。以上这些目标是留有充分余地的，在实际工作中，我们应该奋发有为，把工作做得更好！

为保证各项任务目标的全面完成，集中精力做好以下九项工作：

一、继续深化对于四项重点工作的认识和把握

去年，我们提出了“形成四个共识，突出四个重点，讲究四个策略”的工作思路，在实践中，为进一步突出重点，易于把握，我们又浓缩为“向对外开放求快、向民营经济求活、项目总揽、环境突破”四项重点工作。在这四项工作中，对外开放和民营经济是工作任务，项目总揽是工作措施，环境突破则是工作保障。实践已初步证明，这四项工作确是沧州实现大发展、快发展的关键所在，在认真总结经验的基础上，我们要进一步把这四项重点工作往深里做、往实里做，切实做出新成效。

（一）对外开放务求有实质性突破。无论是实现省委、省政府向我们提出的5到10年再造一个新沧州的目标，还是真正在“两环”开放的大格局中占领一席之地；无论加快沧州工业化的进程，还是尽量缩小同发达地区的差距，我们都必须求助于对外开放。特别是方方面面的条件，已经促成了沧州发展史上对外开放最好的时机，我们一定把握机遇，进一步解放思想，破除对外开放的神秘感，勇于投身对外开放的实践，走出对外开放长期滞后的阴影。首先，迅速整合对外开放的人才队伍。毫无疑问，人才也是对外开放的第一资源，要通过筛选、整合，组建一支有实战能力的专业招商队伍。其次，要把推进优势企业招商引资当作硬任务来完成；要把基础设施、公用事业和第三产业作为利用外资的重要领域。第三，要高起点，敢于问鼎国际国内500强。去年，全市的对外开放工作已经吹响冲锋的号角，既然我们能够突围，我们也一定能够攻占对外开放的高地。对此，全市上下务必形成统一而坚定的信念。

（二）进一步加大固定资产投入特别是工业项目建设力度。历史的经验已经反复证明，投入是沧州经济发展的晴雨表，没有投资的高增长，沧州的经济就很难突出重围，跻身发达地区的行列，我们要紧紧抓住全国新一轮的发展热潮，以项目建设为突破口，寻求沧州经济的大发展和快发展。在整个项目建设中，要突出抓好工业项目，工业项目中既要抓好新上项目，又要重视传统产业升级和企业技术改造项目，而新上项目中则要突出抓好一批立市兴县的大项目。在上大项目这件事上，一定要解放思想、破除迷信，切切实实作为全年项目建设的重点，并务求突破。解放思想、破除迷信的最好办法仍然是走出去看看，一看就可以知道外面是一种什么样的建设局面，就可以明白大项目不是办不到，不是不可为，而是事在人为。在抓项目的同时，要结合城镇建设规划一批工业区域，并积极推动项目进区、集群发展。河间的电线电缆工业区、青县的韩国工业园、盐山的弯头管件工业园、肃宁的裘皮制革工业区、东光的包装机械工业区等10个特色工业区域，要努力完善功能，真正办出特色。

（三）始终把民营经济的发展抓在手上。没有民营经济就没有完善的社会主义市场经济；没有民营经济就没有经济发展的活力；没有民营经济，县域经济就没有支撑和后劲；没有民营经济，就没有我们谋划中的新沧州。发展民营经济，对于我们来说已经不是理论问题，而是实践问题，不是不想发展，更不是限制发展，而是怎样才能发展的快一些。首先要千方百计提供服务，包括政策服务和金融服务等。在政策服务方面，我们出台了那么多鼓励民营企业发展的政策，一定要落到实处，要让民营企业真正享受到政策的实惠；在金融服务方面，要探索通过市场机制，组建担保公司等办法，让民营企业及时得到必要的发展资金。其次，要坚强到位地提供保护。保护民营企业不受各种名目的“检查”和“处罚”的干扰，保护民营企业不受治安环境的威胁，保护民营企业合法的私有财产不受侵犯。当着民营经济成为社会主义市场经济的重要组成部分，当着民营企业家成为有中国特色社会主义事业的建设者，保护他们就是保护和发展先进生产力。

（四）继续强力优化经济发展环境。发展环境的竞争愈来愈激烈，在激烈的竞争中，焦点又是软环境的竞争，也就是优质服务的竞争。今年，要以《行政许可法》的颁布和实施为契机，进一步规范施政行为。真正还权于市场，还权于企业，还权于社会。在减少审批事项和审批环节的同时，还要努力优化服务环境，强化亲商、安商、富商意识，要真正认识到，为客商服务就是为沧州发展服务，为沧州发展服务就是为国家、为民族的繁荣和振兴服务。要进一步深化行风评议活动，并坚决落实一票否决，谁留不住客商，谁就不是一个称职的公务员，谁气走了客商，谁就是沧州发展的罪人。至于对那些欺生排外、欺行霸市的行为，则只有一个选择：狠狠打击，决不手软。

二、毫不动摇地坚持工业强市

工业是经济发展的重要支撑，是财政收入的主要来源，也是第一产业延伸、第三产业繁荣的基础。我市工业虽有一定基础，但起步较晚、总量不大。与先进地区相比，我市经济发展的差距主要在工业，因此，潜力和希望也在工业。工业得不到快速发展，我们面临的许多问题，只能是兵来将挡，水来土掩，而不能从根本上加以解决。一句话，没有高速发展的工业，就没有沧州的富强和明天。我们必须牢固树立工业强市的指导思想，并力促我市现代工业体系的形成。第一，充分发挥优势，谋划工业布局。发展工业，要认真研究我们沧州独特的优势在哪里，然后最大限度地利用这些优势，谋划工业项目。譬如：利用基础设施优势，谋划临港产业，利用地理位置优势，谋划海洋产业，利用自然资源优势，谋划重化工和精细化工等等。第二，培育主导产业，发展县域经济。沧州的发展，说到底是十八个县（市、区）的发展，没有活跃的县域经济，永远不会有富裕的沧州。目前，我市县域经济仍然存在着发展不平衡、特色不鲜明、缺乏足以撑起一县经济的主导产业等问题，针对这些问题，各县（市、区）要从自己的实际出发，对于初具规模的裘皮制革、电线电缆、弯头管件、环保设备、包装机械、新型建材、奶产品加工等产业，通过优势互补、强强联合、资产重组等措施，尽快培育成县域经济的主导产业，同时千方百计壮大龙头企业，带动产业升级，使县域经济既充满活力，又充满特色。第三，推广白庄模式，发展农村工业。白庄模式的根本意义在于它从根本上解决了“三农”问题，基本上消灭了城乡差别和工农差别，而这一切都有赖于它实现了工业化，我们要下大力推广白庄模式。近期内力争每个乡镇都培育起一至两个销售收入超亿元的工业村。第四，大力实施品牌战略。品牌是企业发展的旗帜，而知名品牌，不仅是企业的标识，同时也是一个城市的名片，培育品牌是企业发展的重要战略。运作品牌和运作资金一样，是企业家高超的经营艺术。要千方百计强化企业家的品牌意识，重点扶持一批名牌企业和名牌产品，实现省级著名商标由现有41个增加到56个的目标，并全力以赴实现国家级驰名商标零的突破。通过实施品牌战略，壮大沧州工业的无形资产，并充分发挥品牌效应，在建设强市的同时打造名城。

三、努力加快城镇化进程

加速推进城镇化，是统筹城乡经济社会发展，改变城乡二元结构的重要支点，是促进产业和人口聚集、带动工业化、实现现代化的战略举措。按照“城镇化与工业化、市场化、区域化相结合”的原则，发展壮大中心城市，积极发展中小城市，有重点地发展小城镇，加速推进城乡一体化。第一，突出重点，走大中小城镇协调发展的路子。按照沧州市、县级市、县城、重点建制镇四个层次，分类指导，梯次推进。加快发展壮大沧州市区，把市区放在全省乃至全国大格局中来审视、来定位，放宽视野找对手，提高标准找差距，向最好的看齐，不断提高综合实力，真正发挥在全市城镇化进程中的领跑作用，奠定冀东南中心城市的地位。县级市，加速扩大规模，增强实力，完善功能，力争到2010年发展成为中等城市。县城和重点建制镇建设，立足当地的区位优势，大力发展特色经济，走产业兴城的路子。采取有力措施，引导新上工业项目、新办工业企业、现有企业新上技改项目到统一规划的工业区域兴建，加速生产要素向城镇集中，提高产业聚集度，并促进农业人口向城镇人口转移。力争到2010年，县城平均人口规模达到10万人。第二，抓龙头，充分发挥规划对城镇化发展的指导和调控作用。坚持以新型的发展观为指导，统筹考虑城乡和区域经济发展，进一步深化完善城市总体规划和城镇体系规划，搞好土地修编工作，以科学的规划指导城镇建设，努力做到合理布局，协调发展。严格规划审批制度，强化规划执行，严肃查处违反规划的行为，把各项建设活动全部纳入规划调控范围以内。第三，加快城市基础设施建设，增强综合功能。作为中心城市，沧州市区要建管并重，在抓好新区建设的同时，对老城区实施公厕改造、排水改造、城中村改造、供水管网改造、供暖改造、道路建设、环境建设及清洁城市等事关千家万户切身利益的“八大工程”，其他县级市和县城及中心城镇，也要十分重视基础设施建设，不断为居民创造方便舒适的生活环境和工作环境。

四、稳定增加农民收入，切实做好“三农”工作

坚持以新的发展观指导和做好“三农”工作，以农民增收为目的，以结构调整、科技强农为手段，促进农业农村经济发展。第一，调整结构，做强特色产业，提高农业综合生产能力。坚持从实际出发，按照“特色、规模、品牌、产业化”的思路，积极发展优质、高产、高效、生态、安全农业。大力推进农业和农村经济结构调整，发展奶牛、肉牛100万头；优质牧草100万亩；蔬菜100万亩；无公害果园认证面积达到100万亩；以优质谷子为主的小杂粮100万亩；增加棉花种植100万亩。第二，大力推进农业标准化生产和产业化经营。一是切实抓好农产品质量安全管理。加快农产品质量安全标准与国际接轨，推进农业标准化生产，争创农业特色品牌。重点抓好无公害产品质量认证、良种推广与繁育、动物防疫、饲草饲料、农业技术推广、人工影响天气六大农业社会化服务体系建设。二是发展壮大龙头企业。以培育加工型龙头企业为重点，推动特色产品向优势产区集中，延长产业链条，实现农产品多层次增值。在继续培育壮大小洋人、河英等50家龙头企业的基础上，积极引进市外资金、项目，创建一批新的龙头企业，如天津新佳公司林纸板一体化项目、黄骅新海生物公司水产品开发项目、海兴农业生态科技示范园项目等。三是加强农村合作组织建设，提高农民组织化程度。按照“民有、民营、民享”的原则，重点围绕主导产业，发展农民专业合作经济组织，并引导有能力的专业大户，牵头组建小枣、鸭梨、蔬菜、养牛、草业等专业合作社。第三，加大对农业的投入，努力改善农业生产条件。重点实施“七大工程”：农业开发和土地开发治理工程，争取土地治理规模达到14万亩；脱困工程，力争年内415个贫困村、16.4万贫困人口实现脱贫；“引大入港”工程，确保年底前实现供水；“两渠十库”工程，确保肃宁、争取河间和献县三个水库开工建设；海堤维修加固工程，争取部分项目开工建设；生态家园富民工程，争取国家级生态家园富民工程重点县由2个增加到5个；造林绿化工程，继续加大造林绿化工作力度，加快东部绿化速度，完成八条特色高效绿色示范长廊续建工程，完成两侧造林30万亩。

五、把“四区一园”建设和发展海洋经济作为建设新沧州的支撑点

要想在大发展的热潮中不至于落伍，在激烈的竞争中不被淘汰出局，除去经营好传统经济，使其不断升级换代之外，还要努力培育新的增长区域，不断开拓新的增长领域。

努力做好“四区一园”建设，为在环渤海湾崛起中拥有一席之地奠定新的立足点。举全市之力，加快“四区一园”建设，用最短的时间，将其建设成为全市经济增长的重要支撑力量和吸纳投资的主要基地。临港化工园区，年内开工项目投资50亿元以上，引进项目争取达到20个。确保40万吨PVC、8万吨离子膜烧碱以及配套项目建设，争取5万吨TDI、5万吨/年苯胺、10万吨/年硫酸等项目开工建设。黄骅港开发区，要进一步完善港城规划，年内开工项目投资30亿元，引进项目争取达到20个。重点实施物流中心及仓储、氯酸钾生产、煤制气等超亿元项目。沧州开发区，进一步完善东扩区域基础建设，加快生产力促进中心、高新技术产业园区建设，年内开工项目投资30亿元，引进项目争取达到30个。上述三区要把立足点放在对外开放上，迅速扭转利用外资的被动局面。沧州新区，基础设施建设全面铺开，一批重点项目开工建设。同时，力促神华万亩工业园完成整体规划，并动工兴建基础设施。

大力发展临港产业和海洋经济，为再造一个新沧州夯实新的支撑点。一是加快编制海洋经济发展规划。整合港口、海域、滩涂等优势资源，积极培育海洋生物医药、海水淡化及综合利用等新兴产业。二是以临港工业和物流业为主，大力发展临港产业。包括依托液体化学品码头和杂货码头，重点开发临港化工园区、黄骅港开发区、神华万亩工业园，围绕石油化工、盐化工、精细化工等主导产业，搞好后续产品开发，形成较为完整的化工主导产业群和产品链。同时以建设大型物流基地为核心，发挥港口、铁路、公路交通优势，大力培育现代物流企业，逐步形成辐射中西部地区的物流网络体系。三是催生沿海城市带。按照“以港兴城、以城促港”的方针，港城互动，促使各种要素资源向沿海挺进，加速产业、人口聚集，催生沿海城市带，以城市为依托，滋生沿海经济带。

六、以国企改革为重点，继续深化各项改革

改革创新是加快发展的不竭动力。按照十六届三中全会《决定》的要求，围绕完善市场经济体制，消除妨碍发展的体制性障碍，统筹兼顾，协调推进，力求在以下几个方面实现突破。第一，大力推进国有、集体企业改革。立足“及早改、全面退”，进一步加大国企改革推进力度。积极鼓励和支持外资、民资参与国企改革，推动企业产权多元化。着力抓好沧化集团、沧运集团、化机集团、啤酒厂、制酒厂、造纸厂、机床厂、地铁等一批大中型企业改制方案的落实，力争把所有未改制的企业，包括流通企业改制到位。积极做好“两分”工作，切实减轻企业办社会负担。第二，进一步深化社会保障制度改革。继续巩固“两个确保”，搞好“三条保障线”的衔接。依法扩大养老、医疗、失业保险覆盖面，重点抓好非公有制和灵活就业人员的参保工作。强化社会保险基金征缴，规范基金监管，推进企业退休人员社会化管理服务。做好城市“低保”工作，合理确定最低保障标准和范围。切实做好农村受灾群众生产生活救济工作，在有条件的地方，积极探索建立农村最低生活保障制度。第三，积极稳妥地推进粮食流通体制改革。对全市国有粮食购销企业、附营企业进行产权制度改革，使小型粮食购销企业、附营企业逐步退出“国有序列”。完善农产品市场体系，放开粮食收购市场，落实中央把通过流通环节的间接补贴改为对农民直接补贴的政策，切实保护好种粮农民的利益。

七、大力发展各项社会事业，加强社会主义民主和法制建设

加快科技进步和科技创新。深化科技体制改革，进一步建立和完善有利于促进科技创新、科技进步的体制和机制，加速科技成果向现实生产力转化。探索促进科研院所与企业结合的有效途径，使企业真正成为科研开发和技术创新的主体。在全社会贯彻“尊重劳动、尊重知识、尊重人才、尊重创造”的方针，在各行各业、各个领域建立起有利于人才成长和脱颖而出的机制，加快人才队伍建设。加快培育和建设技术市场，建好科技示范园区，抓好生产力促进中心建设，促进新技术、新成果的广泛应用。整合教育资源，提高教育质量。认真落实全国、全省农村教育工作会议确定的各项政策措施，加快农村教育发展。继续调整教育布局，巩固提高“普九”成果，全面普及九年制义务教育，加快高中阶段教育发展，积极发展高等教育和职业技术教育。启动沧州师专新校区建设，完成沧州卫校新校区一期工程。努力提高卫生工作水平。重点加强农村医疗卫生基础建设，逐步建立新型农村合作医疗制度和医疗救助制度。加强传染病、地方病防治，建立健全城乡疫病预防和公共卫生监控体系，提高人民群众的健康水平。切实加强人口、资源和环境工作。认真做好计划生育工作，逐步建立以奖励救助独生子女家庭为主要内容的利益导向机制，稳定低生育水平，提高人口素质。以循环经济理论为指导，积极开展创建河北省环境保护模范城市和环境优美小城镇活动，抓好污染防治、生态建设与环境保护等重点工作。进一步加强土地利用规划管理，严格执行国家土地政策和有关法规，确保耕地总量动态平衡和耕地占补平衡。积极做好民族宗教工作，大力发展民族经济。关心支持妇女儿童、残疾人事业。搞好国防动员、人民防空及民兵预备役建设，深入开展双拥活动，增进军政军民团结。切实加强精神文明建设。按照发展先进文化的要求，大力弘扬武术、杂技等传统文化，积极引入现代文化。坚持普及与提高相结合，发展群众文化，实施精品战略。大力弘扬奋发有为、勇于创新、乐于奉献、昂扬向上的时代精神，全面提高公民的思想道德素质。深入开展“树文明新风、建文明城市、做文明市民”，以及多种形式的群众性精神文明创建活动，倡导文明健康的生活方式，丰富人民群众的精神文化生活。加强民主法制建设，大力推进依法治市。自觉接受同级人大及其常委会的监督，加强与人民政协的联系。完善了解民情、反映民意、集中民智、珍惜民力的决策机制，建立健全社会公示和专家咨询制度，积极采纳社会各阶层的合理化建议，推进决策科学化、民主化。提高政府法制工作水平，重视行政措施制定的公众参与度，加强行政执法监督，推进依法行政。广泛开展普法教育、法制宣传和法律服务，增强全民法制观念和法律意识。

八、让政府工作更加成熟

坚持以新的发展观指导新时期政府工作，创新思维、创新领导方法和工作方法，不断提高执政本领和工作水平。

第一，准确把握群众脉搏，始终和群众心连心。牢记“两个务必”，大力改进工作作风，深入基层，深入群众，把准群众思想脉搏，掌握第一手材料。健全社情民意反映制度，畅通群众来信来访渠道，及时听取群众呼声。善于站在老百姓的角度去思考问题、部署工作，认真解决关系群众切身利益的“急难盼怨”问题，尽可能多地为人民群众办实事、办好事，提高政府在群众中的威信和凝聚力。对于损害群众利益的事情，要及时发现，及时解决，尽量避免激化矛盾，酿成事端。要永远记住，如果政府是舟，人民群众就是水；如果政府是大树，人民群众就是土壤。

第二，充分发挥后发优势，确保经济健康发展。牢固树立全面、协调、可持续发展的观念，坚持统筹城乡发展、统筹区域发展、统筹经济社会发展、统筹人与自然和谐发展、统筹国内发展和对外开放，促进经济和社会健康发展。在发展中要充分发挥后发优势，尽量少走弯路，避免短期行为，不跟风、不赶潮，一切从沧州实际出发，在加快传统工业由原料型向深加工型升级换代的同时，下决心谋划一批以IT产业为代表的新兴产业和高科技产业，坚决走新型工业化道路。我们虽然对于发展工业如饥似渴，但决不饥不择食。严重伤害群众利益的项目不要；污染严重目前又难以治理的项目不要；暂时赢利但留有后遗症的项目不要。要认真总结并汲取发达地区发展中的经验教训，时刻以清醒的头脑保持沧州经济健康发展。

第三，更加有序地管理城市，营造美好和谐的城市环境。进一步加快政府职能转变，着力解决政府管理经济社会事务中越位、错位、缺位等突出问题，把政府职能转到主要为市场主体服务和创造良好环境上来，加快形成行为规范、运转协调、公共透明、廉洁高效的行政管理体制。不断完善“两级政府、三级管理、四级落实”的城市管理运行机制，充分调动和发挥区、街和社区在城市建设和管理中的作用，推动城市管理重心下移和事权下放，逐步形成市、区、街和社区相互衔接、合理分工和规范高效的城市管理框架，特别要注意发挥社区在城市管理中的作用。同时，运用科学手段，合理配置现代管理设施，加强对市区道路交通、环境卫生、市容市貌、绿化美化的综合整治，让沧州道路畅通，气氛祥和，城市不仅越来越大，而且越来越美。

第四，依法处理各种社会矛盾，有效维护社会稳定。坚持依法办事，正确处理人民内部矛盾，维护群众的合法权益。支持司法机关独立办案。坚持把改革的力度与群众可承受能力有机结合起来，每一项重大政策、措施的出台，事先都要广泛听取社会各界的意见和建议，采纳合理成分，增强科学性和可操作性。同时，还要认真听取司法部门的意见，确保不违法，不违宪。加强对新时期各类复杂社会矛盾的系统研究，找准症结，把握趋向，依法集中解决、攻克一批较深层次的社会矛盾。加大对土地、物权、产权纠纷及其它民间纠纷的调处力度，积极化解人民内部矛盾。始终保持对刑事犯罪的高压态势，加强社会治安防控体系建设，组织开展针对性强的严打整治专项行动，重点打击杀人、抢劫等严重暴力犯罪和入室盗窃、盗抢机动车等多发性侵财犯罪，切实增强人民群众的安全感。

第五，努力提高公务员素质，保证政府廉洁高效运转。遵循“为民、务实、清廉”的原则，切实加强政府自身建设，而政府的形象是所有公务员形象的聚合。对于公务员，坚持凡进必考，把好入口；结合人事制度改革，疏通出口，使公务员队伍成为一池活水，使政府工作充满生机。同时，努力建设学习型政府，利用各种形式，强化公务员的学习和培训，不断提高公务员素质，使广大公务员牢固树立公仆意识，全心全意为人民服务，不失职渎职，不以权谋私。既清正廉洁，忠于职守，又满腔热情，高效施政。

第六，善于应对突发事件，有效保护群众利益。去年一年，连续发生“非典”和风暴潮这样的意外灾害，使我们想起毛泽东主席的一句名言──“人类社会就是在大风大浪中成长起来的”。18个县（市、区）、一万四千平方公里的国土、100公里海岸线，指望年年风调雨顺，天天艳阳高照是不可能的，总会有一些意外突发事件。因此，必须加强对突发事件的预警性研究，建立处置突发事件预警和快速反应机制，分类制定抢险救灾、群体性公共事件、突发事件工作预案，做好应对突发事件的物质和技术准备，最大限度地减少突发事件。而一旦发生突发事件，要确保反应敏捷、处置果断、指挥得当、保障有力，减少突发事件对正常社会生活秩序的冲击，把突发事件造成的损失降到最低限度。要高度重视安全生产工作，强化安全生产监督管理，落实安全生产责任制。加强对重大危险源的监控治理，消除重大事故隐患，避免重大安全生产事故发生。而一旦发生危及人民群众生命安全的突发事件，各级政府一定要靠前指挥，各级领导干部一定要身先士卒，不惜以最大牺牲保护人民群众的利益。

九、振奋精神，建功立业

第一，面对中华民族历史上千载难逢的发展机遇，必须以发展论成败。我们现在正处在历史上最好的发展时期，而这样的时期对于任何一个国家、一个民族来说，都不是经常出现和反复出现的。古今中外，无数事实说明，在历史的机遇面前，不能犹豫彷徨，行动迟缓，更不能熟视无睹，无动于衷。稍一疏忽，失去的不仅仅是机遇，而是一个民族的兴旺和前程。工业革命的机遇被欧美国家抓住了，至今欧美国家仍在领先世界；被满清王朝错过了，导致中华民族长达一百多年落后挨打。因此，我们务必牢记党的十六大关于充分利用本世纪头二十年战略机遇期的论述，增强机遇意识，并紧紧抓住机遇，谋求沧州发展。作为一级政府或政府部门的领导班子，是否胜任，是否优秀，关键看能否抓住机遇，求得发展。可以交学费，但不能永远不毕业；允许摔跟头，但不能永远不前进。不论什么理由，什么动机，在这样大好的机遇面前，事业没有发展，面貌没有改变，即是有辱使命，难辞其咎。因此，当发展成为我们执政兴国的第一要务，而我们又面临千载难逢的发展机遇时，我们必须以发展论成败，以发展论英雄。

第二，沧州历史上为中华民族优秀思想文化和科学技术的发展，作出过突出贡献，今天也一定能够重振雄风、再造辉煌。我们沧州地杰人灵，高适、刘长卿、马致远、纪晓岚……几乎中华民族思想文化发展的每一个重要时期，都闪耀着沧州人的智慧；扁鹊、刘完素、叶圭绶、信都芳、贾耽，和他们紧密相连的是医学、天文、数学、建筑……几乎所有重要科技领域都镶嵌着沧州人的名字。一个历史上对中华民族有着诸多贡献的地区，一个历史上从来不甘寂寞、不甘人后的地区，今天，面对二十年战略机遇期和新一轮发展热潮，我们恰逢天时；环渤海经济带的隆起，黄骅大港的建成，又让我们占尽地利；而全市人民在中共沧州市委的领导下，盼发展的愿望、求发展的热情，又形成了空前的人和和旺盛的人气。有了这一切，我们一定能够不辱祖先，无愧后人。我们一定能够在邓小平理论和“三个代表”重要思想的指引下，紧密团结在以胡锦涛为总书记的党中央周围，用680万勤劳的双手，在华北平原建造起一座经济强市，在渤海之滨托起一颗灿烂新星，一定能够让沧州再一次昂首挺胸走入中华民族复兴的历史史册。
